# Resuscitative endovascular balloon occlusion of the aorta in out-of-hospital cardiac arrest – A Delphi consensus study for uniform data collection

**DOI:** 10.1016/j.resplu.2023.100485

**Published:** 2023-10-11

**Authors:** Helge Haugland, Lorenzo Gamberini, Guillaume L. Hoareau, Matthias Haenggi, Robert Greif, Jostein Rødseth Brede, the REBOA OHCA expert panel and other Collaborators

**Affiliations:** a St. Olav’s University Hospital, Trondheim, Norway; b Norwegian Air Ambulance Foundation, Oslo, Norway; c Department of Anesthesia, Intensive Care and Prehospital Emergency, Ospedale Maggiore Carlo Alberto Pizzardi, Bologna, Italy; d University of Utah, Salt Lake City, USA; e Department of Intensive Care Medicine, Inselspital, Bern University Hospital, Bern, Switzerland; f University of Bern, Bern Switzerland; g School of Medicine, Sigmund Freud University Vienna, Vienna, Austria; h ERC ResearchNet, Niel, Belgium

**Keywords:** Cardiopulmonary Resuscitation, Balloon occlusion, Death, Sudden, Cardiac

## Abstract

**Background:**

Evolving research on resuscitative endovascular balloon occlusion of the aorta (REBOA) as an adjunct treatment for out-of-hospital cardiac arrest mandates uniform recording and reporting of data. A consensus on which variables need to be collected may enable comparing and merging data from different studies. We aimed to establish a standard set of variables to be collected and reported in future REBOA studies in out-of-hospital cardiac arrest.

**Methods:**

A four-round stepwise Delphi consensus process first asked experts to propose without restraint variables for future REBOA research in out-of-hospital cardiac arrest. The experts then reviewed the variables on a 5-point Likert scale and ≥75% agreement was defined as consensus. First authors of published papers on REBOA in out-of-hospital cardiac arrest over the last five years were invited to join the expert panel.

**Results:**

The data were collected between May 2022 and December 2022. A total of 28 experts out of 34 primarily invited completed the Delphi process, which developed a set of 31 variables that might be considered as a supplement to the Utstein style reporting of research in out-of-hospital cardiac arrest.

**Conclusions:**

This Delphi consensus process suggested 31 variables that enable future uniform reporting of REBOA in out-of-hospital cardiac arrest.

## Introduction

Resuscitative endovascular balloon occlusion of the aorta (REBOA) is a potential therapeutic adjunct in managing hemorrhagic shock in trauma.^1^, ^2^ REBOA consists of introducing an aortic balloon catheter through an arterial femoral access, and once the appropriate position is reached, the balloon is inflated to occlude the aorta.^1^, ^3^ Hence, the result is a redistribution of cardiac output to the organs proximal to the occlusion, overall heart and brain, and a reduction of distal blood flow.

For REBOA deployment, the aorta is functionally divided into three zones.^1^, ^4^ The appropriate occlusion zone depends on the bleeding source, with Zone 3 proposed for exsanguinating pelvic, lower junctional and lower limbs hemorrhages. In contrast, Zone 1 is suggested for abdominal exsanguination and impending cardiac arrest.^5^, ^6^ REBOA is also used to manage hemorrhagic shock from non-traumatic aetiology, such as aortic aneurysm rupture, gastrointestinal bleeding, or obstetric hemorrhage, particularly postpartum hemorrhage.^7^, ^8^, ^9^, ^10^

More recently, preclinical studies suggested that REBOA increases coronary and cerebral blood flow during resuscitation.^11^, ^12^, ^13^, ^14^, ^15^ Aortic occlusion is therefore advocated as a potential adjunct to cardiopulmonary resuscitation (CPR) to treat non-traumatic cardiac arrest.^16^, ^17^ The feasibility of REBOA during CPR in traumatic and non-traumatic cardiac arrest is demonstrated in observational studies, with balloon deployment either in the emergency department or pre-hospital setting.^18^, ^19^, ^20^, ^21^, ^22^, ^23^, ^24^, ^25^, ^26^ Currently, randomized controlled trials assess the efficacy of REBOA as an adjunct treatment in traumatic hemorrhage^27^ and in non-traumatic out-of-hospital cardiac arrest (OHCA).^28^

The use of REBOA as an adjunct treatment in OHCA is evolving but still not widespread. The heterogeneity of published results in studies on REBOA calls for uniform reporting of the REBOA-related variables. Such a standard set of variables will allow registration and reporting of the same variables, complementing the commonly used Utstein resuscitation registry template.^29^, ^30^ This may increase data quality and enable future comparison of studies and systematic reviews with meta-analysis. The study aimed to establish a set of variables related to REBOA in OHCA through a Delphi consensus process of international experts in the field.

## Methods

### Study design

The standard set of variables was established through a Delphi technique consensus process.^31^

### Research question

We hypothesized that different stakeholders would have a range of opinions on which variables to collect in research on REBOA in OHCA. Therefore, our research question was: “Which variables should be included in a template for data collection and reporting in future research on REBOA in OHCA?”.

### Data collection and management

We used a four-round Delphi study using expert panel consensus. The technique is based on a panel of experts in the field, who are asked to answer questions or decide on statements based on their opinions and judgments on a defined topic. The Delphi technique has successfully obtained consensus in emergency care through successive surveys – often called “rounds”.^32^, ^33^, ^34^ An important principle is that an expert's response will be anonymous to the other experts. I.e., experts will be presented with the proposals from the other group members, but who proposed what will not be revealed at any point. Thus, it is essential that experts do not discuss the selection of variables with other experts during the study.

We followed the Guidance on Conducting and Reporting Delphi Studies (CREDES).^35^ The checklist is available as supplementary material.

The study consisted of the following four e-mail rounds to the experts:

In round 1, the experts were asked to propose which REBOA-related variables should be included in a common data set for future research on REBOA in OHCA. After the first round, the proposals were read and edited by one of the authors who did not participate in the expert panel; i.e. an independent methodologist (HH). Moreover, similar answers were merged to compile the second round of questions.

In round 2, the experts were asked to rate agreement or not with all the proposals using a 5-point Likert scale (1; strongly disagree, 2; moderately disagree, 3; neither agree nor disagree, 4; moderately agree and 5; strongly agree). After that, the median and interquartile range (IQR) and the percentage of agreement for each statement were calculated by adding the 4 (moderately agree) and 5 (strongly agree) ratings, calculating the proportion of the total number of answers for each statement. The pre-defined cut-off for consensus was 75% agreement and a median score of 5. Items with consensus in round 2 were qualified to be part of the final list of REBOA-related variables. Items with disagreement (median ≤ 3) were excluded at this point.

Round 3 showed the experts the results from round 2 (median and level of agreement for each statement). Proposals which were neither agreed nor disagreed upon in round 2 were re-rated by all experts who could only respond “yes” or “no” to whether the remaining proposals should be part of the final list of REBOA-related variables. Variables with ≥ 75% expert agreement were included.

Finally, in round 4, the list of variables was e-mailed to the experts for their enclosing comments and approval.

### Study participants

The core study group (HH, LG and JRB) identified authors who had published manuscripts about REBOA in non-traumatic or traumatic OHCA in humans and animal models from 2017 to 2021. We invited the first or corresponding authors or another co-author recommended by the first author, which resulted in 31 authors being invited. Moreover, the study group could nominate up to three additional experts based on their expertise in the field. Finally, 34 experts were invited. The experts received only information about the four steps of the consensus process.

## Results

Of the 34 invited experts, 30 (88%) accepted the invitation and joined the expert panel. The data were collected between the 6th of May 2022 and the 19th of December 2022, over 30 days in each round. In addition, up to two reminder e-mails were sent to experts who did not respond within the designated deadline.

### First round results

**Fig. 1:**
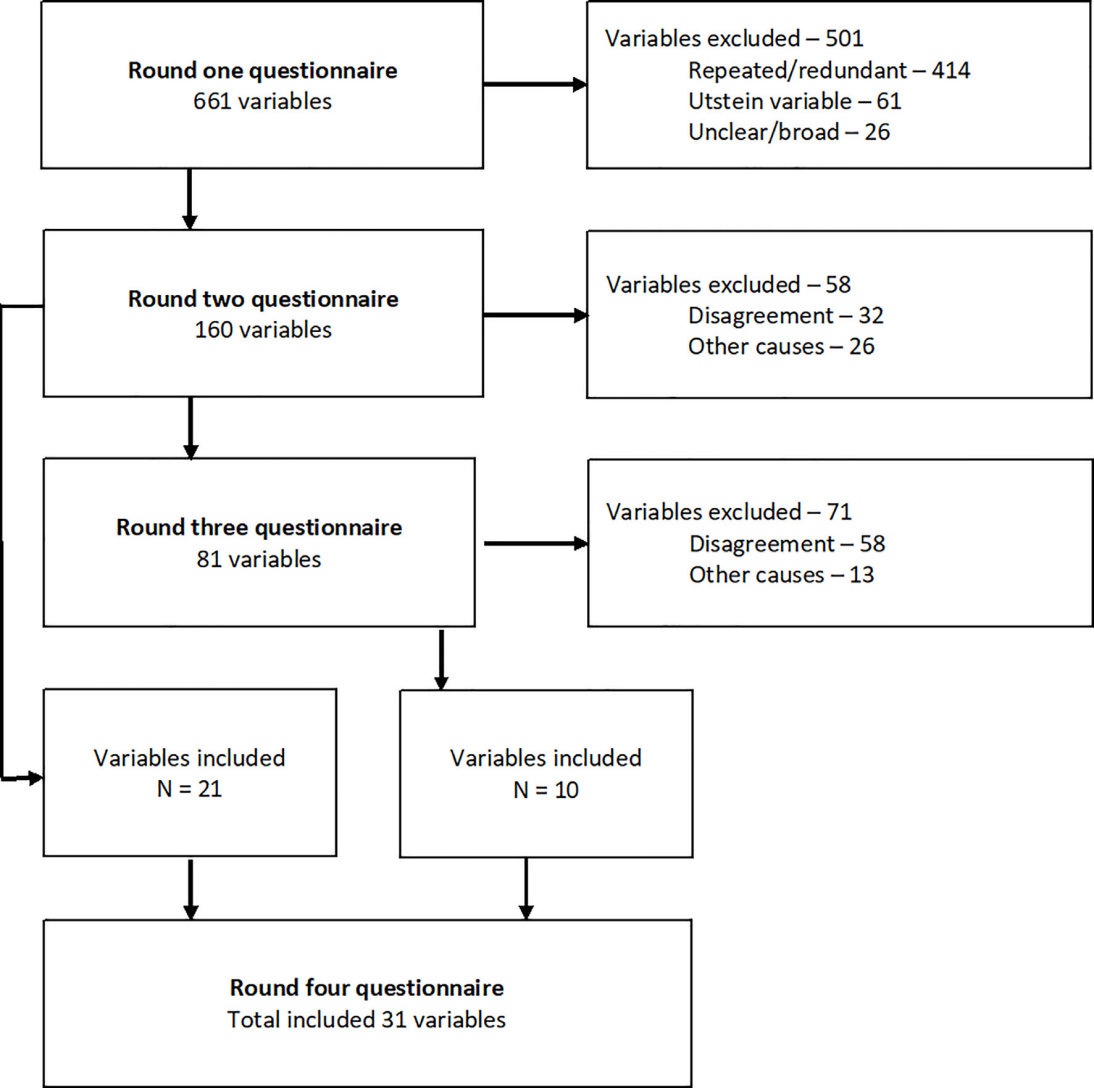
Flowchart of variables in the Delphi rounds.

Round one enrolled 29 (97%) of the 30 experts, which were asked to propose up to 10 relevant variables in the following framework of categories; system, patient characteristics, process, outcome, and other variables if a proposal did not fit into the four categories. The experts proposed 661 variables. Similar variables were merged, and redundant variables, poorly described or outside the scope of this study, were excluded (Fig. 1).

### Second round results

There was one dropout in round two (response rate 97%). Twenty-one variables with ≥75% agreement and a median rating of 5 were accepted as consensual and did not enter round three. Items with a median rating of ≤ 3 were excluded and did not enter round three. Eighty-one variables did not fulfill the exclusion or inclusion criteria and were entered to round three for re-rating.

### Third round results

There was no dropout in round three (response rate 100%). Thus, a total of 28 experts completed all three rounds. In this round, 10 variables reached consensus and were included in the final variable set, which consists of 31 variables that are presented in Table 1.

**Table 1:** Final set of variables for studies on REBOA in out-of-hospital cardiac arrest as addition to standard Utstein data reporting system.

**No**	**Variable name**	**Explanation of variable (if necessary)**
**System variables - to be registered once before study start**		
1	Availability of service	When is REBOA available, e.g., 24/7, daytime, 5 days a week
2	REBOA team composition	e.g., physician, paramedic, nurse
3	What is the REBOA training for providers?	Formal certification Yes/No, Formal re-certification Yes/No
4	Existing description of REBOA procedure?	Guideline, standard operating procedure
5	Description of inclusion and exclusion criteria	Describe inclusion and exclusion criteria

**Patient variables**		
6	If trauma: Injury Severity Score	
7	Haemorrhage as cause of arrest	Yes/No
8	Anticoagulation medication	Yes/No. If yes: type of anticoagulation medication

**Process variables**		
9	Setting of REBOA procedure	Pre-hospital or in-hospital deployment
10	Profession of REBOA operator	1. Physicians specialty (Emergency physician, anaesthesiologist, intensive care doctor, surgeon, radiologist, other) 2. Paramedic 3. Nurse. 4. Other
11	Time from dispatch of unit until start of REBOA procedure	Start of procedure is the first cannulation attempt.
12	Time from dispatch of unit until inflation of balloon	
13	Time from REBOA balloon inflation to deflation (if any)	Duration REBOA was inflated
14	Time from aortic occlusion to ROSC	Time from balloon inflation to ROSC (if any).
15	Cannulation technique	Ultrasound guided, blind or surgical
16	Number of cannulations attempts to establish femoral access	
17	REBOA catheter model used	Catheter type
18	Introducer sheath size	In French
19	Verification of arterial positioning	E.g., ultrasound, fluoroscopy
20	Was REBOA procedure completed?	Yes/No. If no; report cause
21	REBOA zone	Zone I-II-III
22	Total number of balloon inflations	
23	End tidal CO_2_ pre-balloon	
24	End tidal CO_2_ after occlusion	Preferably in time intervals of 30 s, 60 s, 90 s, 120 s after occlusion
25	Blood pressure proximal to occlusion, if available	Before REBOA, 1 min and 5 min after REBOA, and after ROSC
26	Registered cardiac rhythm	Prior to REBOA inflation and 1, 3 and 5 minutes after inflation
27	Did REBOA procedure negatively influence resuscitation quality?	Yes/No

**Outcome variables**		
28	Numbers of “any ROSC” episodes	Any ROSC is ROSC regardless of duration
29	Sustained ROSC	ROSC lasting 20 minutes or longer
30	Vascular access complications	1. Infection at vascular access site requiring antibiotics 2. aortic injuries 3. retroperitoneal hematoma 4. other complications

**Other variables**		
31	REBOA equipment used for access to ECMO or PCI?	Yes/No

Abbreviations: REBOA - resuscitative endovascular balloon occlusion of the aorta; ROSC - return of spontaneous circulation; ECMO - extracorporeal membrane oxygenation; PCI - percutaneous coronary intervention.

Notes: System variables are only to be registered once (before study start). The other variables are to be registered after each REBOA procedure.

### Fourth round results

All experts approved the final variable set. Two experts emphasized the importance of registering mortality after hospital admission, such as survival to discharge or 30 days mortality, for patients included in future REBOA studies.

The geographical distribution of the experts completing all the rounds is shown in Table 2.

**Table 2:** Geographical distribution of the experts’ participating institutions.

Country	Experts
Canada	1
China	1
France	1
Germany	1
Italy	3
Japan	1
Norway	5
Sweden	3
Switzerland	2
United Kingdom	1
USA	9

## Discussion

Previous work has tried to establish consensus on uniform variables in other areas of REBOA use, such as indications, contraindications, patient selection in traumatic and non-traumatic hemorrhage, a core outcome set, and developing a tool for assessing procedural competence.^36^, ^37^, ^38^

Due to the increasing interest in REBOA applications in both traumatic and non-traumatic OHCA, we aimed to achieve an international expert consensus on the system-, patient-, process- and outcome related variables to be registered in future studies on REBOA use in OHCA. The objective is to get more uniform study designs for collected variables and enable data comparability for future studies.

The role of aortic occlusion still needs to be defined in relation to the increasing number of other advanced therapeutic options available in both traumatic and non-traumatic cardiac arrest. Most of the 31 variables suggested are process variables that may reflect a need to increase knowledge of the technical aspects of the REBOA procedure, such as location, timing, positioning, and verification modalities. Furthermore, this study highlights the demand for clinical data on how REBOA influences both non-invasive and invasive cardiac output calculations, organ perfusion during CPR, and the evolution of cardiac arrest rhythms before and after balloon occlusion.

The main theoretical mechanism of action for REBOA during CPR is a selective increase in coronary and cerebral perfusion pressure^39^. Animal models suggest that not only the coronary perfusion pressure threshold but also its dose, defined as the area under the curve of coronary perfusion pressure during resuscitation as an estimate of total measured perfusion, strongly influences return of spontaneous circulation (ROSC) probability.^40^ Hence, both aortic occlusion per se and time from collapse to aortic occlusion could strongly influence the potential effect of REBOA on ROSC probability. Therefore, early pre-hospital REBOA procedure rather than awaiting hospital arrival may improve outcomes. Moreover, the underlying OHCA etiology may influence the response to aortic occlusion; hence a subgroup assessment of different cardiac rhythms and possible etiology is warranted. Cerebral perfusion pressure, which determines a good neurological outcome if the patient survives, may be positively affected by REBOA during CPR. Experimental data demonstrate increased cerebral perfusion pressure.^14^ Human data may never be collected, as intracranial pressure measurement cannot be done during cardiac arrest. Single patient data of brain tissue near-infrared spectroscopy as a surrogate marker of brain perfusion point towards improved brain perfusion.^21^

The REBOA procedure may interact negatively with the quality of the CPR provided, such as focus shift of the providers, interruption of compressions, increase in hands off time. Hence, the main variables related to resuscitation quality suggested by contemporary guidelines should be strictly monitored and reported. Recent studies report that education in REBOA catheter positioning through blended courses is feasible regardless of pre-existing vascular access skills, also for cardiac arrest scenarios.^41^, ^42^

A common set of variables may only be useful if researchers indeed know the recommended variable set. Hence, this Delphi consensus will be proposed to different international scientific societies interested in REBOA and endovascular resuscitation to promote its dissemination and foster adherence to the proposed variables.

### Strengths and limitations

This study included experts on REBOA in cardiac arrest from both human studies and animal models. Moreover, the experts were recruited from different countries, making it an international expert panel. There is no consensus on panel size; however, our panel size of 28 is within the recommendations of 15–30 for heterogeneous groups.^43^

The data collection was time-consuming, and reminders to the experts were necessary. Completing three e-mail rounds, and making their own proposals in the first one, is a substantial effort and calls for time and motivation with the experts, which could mean that only the most motivated experts would participate. Despite the Delphi process being an effective method for reaching a consensus on complex healthcare questions, the definition of consensus is not uniform. Studies report levels of consensus differing from 51%–80%.^35^, ^44^ Thus, our a-priori definition of consensus seems adequately related to previous studies. Even for the variables where consensus was reached, this does not necessarily reflect the one and only “truth”, as a differently composed expert panel could have reached a consensus on other variables. Moreover, it is well known that the Delphi process tends to eliminate extreme opinions and rather lead the experts to be more conservative when trying to reach a consensus.^45^

## Conclusion

In this international Delphi consensus study, we present 31 suggested variables to be collected in future studies on REBOA in out-of-hospital cardiac arrest. These core variables will complement the Utstein cardiac arrest reporting systems and allow comparison of studies in systematic reviews with meta-analysis.

## Ethics approval and consent to participate

This study followed the Helsinki Ethical Principles for Medical Research Involving Human Subjects. Ethics committee approval and informed consent were not needed for this study due to its nature (Delphi Consensus). All questionnaires were accompanied by written information that explained the purpose of each round. All the Authors and Collaborators approved the final manuscript.

## Funding

No external funding was received for this study.

## Availability of data and materials

The datasets analyzed during the current study are available from the corresponding author on reasonable request.

## CRediT authorship contribution statement

**Helge Haugland:** Conceptualization, Methodology, Validation, Formal analysis, Data curation, Writing – original draft. **Lorenzo Gamberini:** Conceptualization, Formal analysis, Writing – original draft, Supervision. **Guillaume L. Hoareau:** Conceptualization, Writing – review & editing. **Matthias Haenggi:** Conceptualization, Writing – review & editing. **Robert Greif:** Conceptualization, Writing – review & editing. **Jostein Rødseth Brede:** Conceptualization, Formal analysis, Writing – original draft, Supervision.

## Declaration of competing interest

The authors declare the following financial interests/personal relationships which may be considered as potential competing interests: [Guillaume L. Hoareau is a shareholder of Certus Critical Care. Robert Greif is ERC board director of Guidelines and ILCOR, and chair of ILCOR’s Task Force Education, Implementation and Team. M. Austin Johnson is a founder of Certus Critical Care, Inc. Craig D. Nowadly worked as an independent contractor for Certus Critical Care, a relationship that concluded in 2020. Wolf E. Hautz has received research funding from the European Union, the Swiss National Science foundation, Zoll foundation, Dräger Medical Germany, Mundipharma Research UK, MDI International Australia, Roche Diagnostics Germany, all outside the submitted work. He has provided paid consultancies to AO foundation Switzerland, MDI International Australia, and SIWF, all outside the submitted work. Finally, he has received financial support for a congress he chaired from EBSCO Germany, Isabel Healthcare UK, Mundipharma Medical Switzerland, VisualDx USA, all outside the submitted work. Federico Semeraro is the Chair-Elect of the European Resuscitation Council, Chair of the ILCOR Social Media Working Group and ILCOR BLS Working Group members.].

## Appendix 1

REBOA OHCA expert panel and other Collaborators

The study group would like to thank the following members of the “REBOA OHCA expert panel” for their valuable participation: Craig D. Nowadly (USA), Philippe Rola (Canada), Jacob Glaser (USA), Byron Drumheller (USA), David T. McGreevy (Sweden), Carlo Alberto Mazzoli (Italy), Jo Kramer-Johansen (Norway), Ryo Yamamoto (Japan), Tal Hörer (Sweden), Wolf Hautz (Switzerland), Pål Klepstad (Norway), Mohamad Hakam Tiba (USA), Emanuel Dogan (Sweden), Peter Hilbert-Carius (Germany), M. Austin Johnson (USA), Andreas Krüger (Norway), Trond Nordseth (Norway), Jiefeng Xu (China), Carlo Coniglio (Italy), James Daley (USA), William A. Teeter (USA), Anja Levis (Switzerland), Alice Hutin (France) and Sharaf Khan (USA).

The study group would also like to thank Federico Semeraro (Italy) for participating in the early planning of the study.
